# Patients double-seropositive for ANCA and anti-GBM antibodies have varied renal survival, frequency of relapse, and outcomes compared to single-seropositive patients

**DOI:** 10.1016/j.kint.2017.03.014

**Published:** 2017-09

**Authors:** Stephen P. McAdoo, Anisha Tanna, Zdenka Hrušková, Lisa Holm, Maria Weiner, Nishkantha Arulkumaran, Amy Kang, Veronika Satrapová, Jeremy Levy, Sophie Ohlsson, Vladimir Tesar, Mårten Segelmark, Charles D. Pusey

**Affiliations:** 1 Renal and Vascular Inflammation Section, Department of Medicine, Imperial College London, London, UK; 2 Department of Nephrology, General University Hospital, Prague and First Faculty of Medicine, Charles University, Prague, Czech Republic; 3 Department of Nephrology and Transplantation, Skånes University Hospital, Lund, Sweden; 4 Department of Nephrology and Department of Medical and Health Sciences, Linköping University, Linköping, Sweden

**Keywords:** anti-GBM disease, anti–neutrophil cytoplasm antibody, glomerulonephritis, Goodpasture syndrome, vasculitis

## Abstract

Co-presentation with both ANCA and anti-GBM antibodies is thought to be relatively rare. Current studies of such ‘double-positive’ cases report small numbers and variable outcomes. To study this further we retrospectively analyzed clinical features and long-term outcomes of a large cohort of 568 contemporary patients with ANCA-associated vasculitis, 41 patients with anti-GBM disease, and 37 double-positive patients with ANCA and anti-GBM disease from four European centers. Double-positive patients shared characteristics of ANCA-associated vasculitis (AAV), such as older age distribution and longer symptom duration before diagnosis, and features of anti-GBM disease, such as severe renal disease and high frequency of lung hemorrhage at presentation. Despite having more evidence of chronic injury on renal biopsy compared to patients with anti-GBM disease, double-positive patients had a greater tendency to recover from being dialysis-dependent after treatment and had intermediate long-term renal survival compared to the single-positive patients. However, overall patient survival was similar in all three groups. Predictors of poor patient survival included advanced age, severe renal failure, and lung hemorrhage at presentation. No single-positive anti-GBM patients experienced disease relapse, whereas approximately half of surviving patients with AAV and double-positive patients had recurrent disease during a median follow-up of 4.8 years. Thus, double-positive patients have a truly hybrid disease phenotype, requiring aggressive early treatment for anti-GBM disease, and careful long-term follow-up and consideration for maintenance immunosuppression for AAV. Since double-positivity appears common, further work is required to define the underlying mechanisms of this association and define optimum treatment strategies.

Anti-glomerular basement membrane (GBM) disease and the anti-neutrophil cytoplasm antibody (ANCA)-associated vasculitides (AAV) are rare conditions, with estimated incidences in Europe of 1 and 20 per million population per year, respectively.^1^, ^2^ The concurrence of both ANCA and anti-GBM antibodies in individual patients, however, is well-recognized, and occurs at a much higher frequency than would be expected by chance alone. This phenomenon was first reported within a few years of the first description of ANCA in the 1980s,^3^, ^4^ and has been observed in several series from around the world over the subsequent 30 years.^5^, ^6^, ^7^, ^8^ It is clear that the 2 antibody populations associated with these diseases are antigenically distinct,^9^ and that this phenomenon is not due to cross-reactivity, although the mechanisms of the association are not fully understood.

Several studies have reported the outcomes of these patients who are double positive, although with conflicting findings; some have observed better outcomes compared with those with single-positive anti-GBM disease,^4^, ^10^, ^11^ while others have suggested that patients who are double positive have comparable or worse outcomes.^5^, ^6^, ^12^, ^13^, ^14^, ^15^, ^16^ These studies, however, have generally been limited by small size (many describing fewer than 20 cases) and variations in the severity of disease at presentation, with between 0% and 100% of patients being dependent on dialysis at diagnosis.^8^, ^15^ Furthermore, in the largest series to date, from Chinese centers, fewer than 25% of patients were treated with plasma exchange, and so the applicability of the findings to European patients treated with substantially different therapeutic regimens is limited.^7^, ^16^

The aim of the present study is to describe the clinical features and long-term outcomes of a contemporary cohort of patients with double-positive ANCA and anti-GBM disease. Given the rarity of these patients, we have identified cases from 4 large Northern European nephrology centers, which employ comparable treatment protocols for these cases, including plasma exchange, cyclophosphamide, and steroids, unless contraindicated. We have compared clinical features and outcomes to those for single-positive AAV and single-positive anti-GBM disease. Because patients with double-positive disease more closely resemble those with single-positive anti-GBM disease at presentation, we have also compared histopathology and treatment in these 2 groups.

## Results

### Case identification and demographics

**Figure 1:**
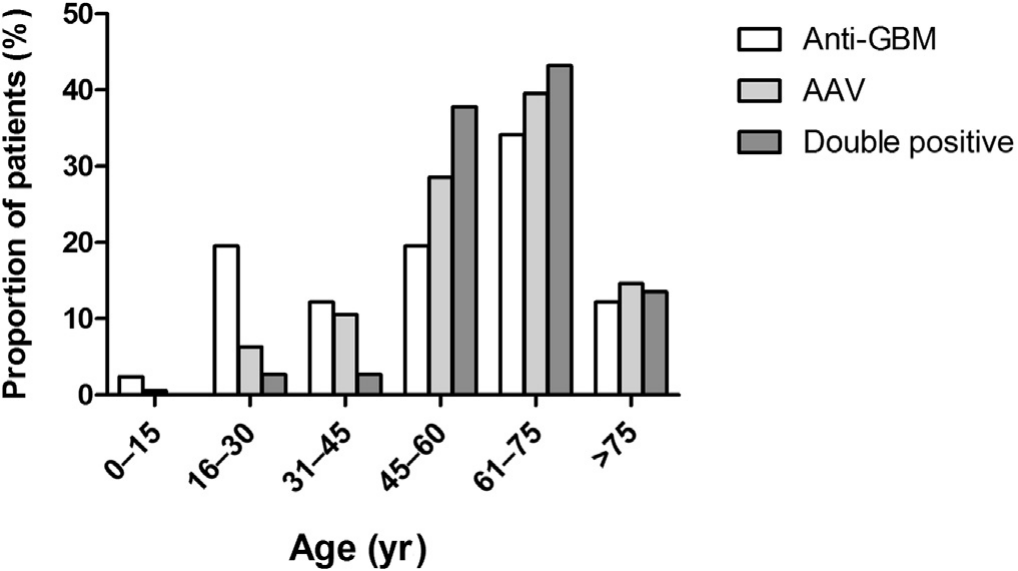
**Age distribution of patients with anti–glomerular basement membrane (GBM) disease, anti-neutrophil cytoplasm antibody–associated vasculitis (AAV), and double positive disease at presentation**.

Between 2000 and 2013, a total of 646 cases were identified at 4 centers in 3 countries, including 568 patients with single-positive AAV, 41 with single-positive anti-GBM disease, and 37 patients who were double positive for anti-GBM antibodies and ANCA (hereafter AAV, anti-GBM, and double-positive groups, respectively) (Table 1). The ratio of double-positive to single-positive anti-GBM cases was similar in all 3 countries (47% overall); however, patients who were double positive represented a variable proportion of the AAV cases (3% to 10.5%; 6.1% overall). The demographic features of the cohort are summarized in Table 1. The single-positive anti-GBM group demonstrated the typical bimodal age distribution of this disease, whereas patients who were double positive had an age distribution similar to patients with isolated AAV (Figure 1). There was no significant difference in gender ratio between the 3 groups. Notably, 1 patient in the double-positive group had a previous diagnosis of isolated anti-myeloperoxidase (MPO) and AAV 2 years prior to presenting with double-positive disease.

**Table 1:** Case identification, demographics, clinical features, and serology

	AAV	Anti-GBM	Double positive	*P* value			
				AAV versus DP versus GBM	AAV versus DP	GBM versus DP	AAV versus GBM
Cases, *n*	568	41	37	–	–	–	–
• United Kingdom	171	19	20				
• Sweden	100	13	8				
• Czech Republic	297	9	9				
Cases, %	87.9%	6.3%	5.7%				
**Demographics**							
Age, yr (range)	62.3 (11–95)	58.3 (13–91)	63.6 (17–88)	0.17	0.99	0.31	0.21
Gender							
• Male	54%	46%	38%	0.11	0.06	0.49	0.34
• Female	46%	54%	62%				
**Clinical Features**							
Duration of symptoms,^a^ wk (range)	12 (0–56)	2 (0–20)	10 (1–26)	**<0.01**	0.99	**<0.01**	**<0.01**
Lung hemorrhage	131/568 23%	16/41 40%	14/37 38%	**0.01**	**0.04**	0.85	**0.02**
Required RRT at presentation	132/568 23%	26/41 63%	21/37 57%	**<0.01**	**<0.01**	0.55	**<0.01**
eGFR,^b^ ml/min (range)	29 (5–90)	20 (5–90)	19 (6–76)	0.06	0.11	0.99	0.67
Serum creatinine,^b^ μmol/l (range)	186 (39–693)	275 (62–667)	309 (71–606)	0.06	0.18	0.99	0.37
**Serology**							
Anti-GBM level, xULN (range)	–	5.4 (1–29.1)	14.2 (1–50.4)		–	0.06	–
Proportion seronegative for anti-GBM, %	–	4/41 11%	4/37 11%		–	1.00	–
ANCA serology, %					**<0.01**	–	–
• Anti-MPO	48%		70%				
• Anti-PR3	51%		27%				
• Anti-MPO & PR3	<1% (*n* = 2)		3%				

AAV, anti-neutrophil cytoplasm antibody–associated vasculitis; DP, double-positive; eGFR, estimated glomerular filtration rate; GBM, glomerular basement membrane; MPO, myeloperoxidase; PR3, proteinase 3; RRT, renal replacement therapy; xULN, multiples of upper limit of normal.

Results expressed as median ± range. Comparison between groups by Kruskall–Wallis test with Dunn’s post-test to ascertain differences between individual groups (for continuous data), or by chi-square test (for categorical data).

a Calculated for a sample of 48 ANCA cases.

b Censored for patients on RRT.

### Clinical presentation and serology

Table 1 summarizes key clinical features and serological findings at presentation. The duration of symptoms prior to receiving a diagnosis was similar in the AAV and double-positive groups (median: 10–12 weeks), and this was significantly longer than in single-positive anti-GBM patients (2 weeks; *P* < 0.01). Despite shorter duration of symptoms, the severity of disease—whether defined as need for hemodialysis or, in patients who were not dialysis-dependent, by GFR estimations or serum creatinine measurements—in anti-GBM patients was similar to that of patients who were double positive. The frequency of alveolar hemorrhage was similar in anti-GBM and double-positive groups, occurring in about one-third of patients. Severe disease manifestations (dialysis requirement and lung hemorrhage) were less common in patients with AAV, each occurring in approximately one-quarter of cases. Patients who were double positive had additional extrarenal manifestations, including nonhemorrhage lower respiratory tract disease (in 26%), otorhinolaryngological involvement (18%), musculoskeletal symptoms (18%), cutaneous features (13%), neurological (8%), gastrointestinal (5%), and ocular symptoms (3%).

Because a variety of anti-GBM assays were used during the study period and at different hospital sites, results were standardized by expressing them as multiples of the upper limit of normal (xULN) for each assay. Patients who were double positive tended to have lower levels of circulating anti-GBM antibodies than did anti-GBM patients who were single positive, although the difference between groups was not statistically significant. A similar proportion of patients in both these groups (approximately 10%) were seronegative for circulating anti-GBM antibodies. In these cases, there was convincing evidence of linear IgG deposition on renal biopsy, in the absence of another attributable cause, in keeping with our definition of anti-GBM disease.

In the AAV group, the proportion of patients who were positive for anti-proteinase 3 (PR3) versus anti-MPO antibody was approximately equal. However, in the double-positive groups there was a comparative over-representation of patients with anti-MPO antibodies (70% vs. 48%, *P* < 0.01). One patient in the double-positive group was triple positive for anti-MPO, anti-PR3, and anti-GBM antibodies. Notably, this patient had a history of recreational drug use and was positive for hepatitis C virus. Because a variety of methodologies, including indirect immunofluorescence and antigen-specific assays, were used to confirm ANCA positivity over the period of the study, it was not possible to standardize comparisons of ANCA titer.

### Histopathology

Because the anti-GBM and double-positive groups had similar disease severity at presentation, we performed more detailed analysis to compare histopathological and therapeutic differences in these 2 cohorts. Approximately two-thirds of patients in both groups underwent renal biopsy, as described in Table 2. The severity of renal disease in those who underwent biopsy was similar in both groups and equivalent to the severity of renal disease in the parent cohort, suggesting that biopsy findings may be representative and comparable between groups. It was not possible, however, to retrospectively identify the reasons why renal biopsy was not undertaken in all remaining patients. In some cases, this was due to the need for immediate treatment with plasma exchange, and others were considered too clinically unstable for biopsy. In keeping with the age difference between the parent groups, the mean age in the subset of patients who underwent biopsy was lower in the anti-GBM group compared with the double-positive group.

**Table 2:** Histopathology

	Anti-GBM	Double positive	*P* value
Underwent biopsy, n (%)	29 (71%)	25 (68%)	0.81
Mean age at biopsy, yr (range)	46 (13–91)	62 (46–76)	**<0.01**
Renal status at biopsy			
• Required RRT	52%	54%	1.00
• eGFR,^a^ ml/min (range)	21 (5–90)	16 (8–73)	0.78
• Serum creatinine^a^ μmol/l (range)	275 (62–677)	315 (71–606)	0.75
Glomerular findings			
• Crescentic glomeruli, %	64% (0–100)	64% (25–100)	0.98
• Sclerotic glomeruli, %	0% (0–80)	15% (0–100)	0.19
• Normal glomeruli, %	5% (0–100)	0% (0–67)	0.56
Tubular atrophy, % (range)	5% (0%–30%)	27% (0%–80%)	**<0.01**
Immunofluorescence pattern			0.69
• Linear IgG	79%	80%	
• Pauci-immune	3%	8%	
• Technically inadequate	17%	12%	

eGFR, estimated glomerular filtration rate; GBM, glomerular basement membrane; RRT, renal replacement therapy.

Results expressed as median ± range. Comparison between groups by Mann-Whitney test (for continuous data), or by chi-square test (for categorical data).

a Censored for patients on RRT.

The median number of glomeruli in each biopsy was 15 (interquartile range: 10–20). There was no difference in the proportion of crescentic glomeruli between the 2 groups. There was, however, a tendency for more sclerotic glomeruli to be observed in patients who were double positive (median: 15% vs. 0%; *P* = 0.188). Likewise, the finding of “synchronous” crescent formation tended to be more commonly observed in patients with anti-GBM disease (73% vs. 33% in patients who were double positive; *P* = 0.092). There was a highly significant difference in the degree of interstitial fibrosis and tubular atrophy between these 2 groups, with more evidence of chronic damage in double-positive cases (median: 27% vs. 5%, *P* < 0.001). In those cases in which adequate tissue was available for analysis, all but 3 cases (2 double-positive, 1 single-positive for anti-GBM, and all with circulating anti-GBM antibodies) had linear deposition of IgG by immunofluorescence analysis of the kidney biopsy.

### Treatment

There was no detectable difference between single-positive anti-GBM and double-positive groups with regard to initial treatment administered, the majority receiving standard of care with steroids (97% vs. 100% in double-positive and anti-GBM cases, respectively; *P* = 0.47), cyclophosphamide (100% vs. 92%; *P* = 0.24), and plasma exchange (80% vs. 89%; *P* = 0.33). In total, 10 patients did not undergo plasma exchange for various reasons. In the double-positive group, 7 patients did not receive plasma exchange. Of these, 2 were dialysis dependent at presentation with 100% crescent formation on kidney biopsy, and in the absence of lung hemorrhage, plasma exchange was deemed futile. These patients received cytotoxic therapy and steroids for nonrenal manifestations. Of the other 5 patients, none had alveolar hemorrhage or required dialysis, and their initial treatment was typical of those presenting with isolated AAV. In the single-positive anti-GBM group, 1 patient was dialysis dependent with no normal glomeruli on kidney biopsy, and so treatment was deemed futile; 1 patient had well-preserved renal function (serum creatinine: 78 μmol/l) and so plasma exchange was initially reserved for nonresponse to immunosuppressive treatment alone, and was ultimately not required; and 1 patient was clinically unstable and therefore unable to undergo plasma exchange.

At 6 months, 74% of patients who were double positive were receiving ongoing immunosuppressive treatment with or without corticosteroids (71% with azathioprine, 21% with mycophenolate mofetil, and 8% with methotrexate), whereas only 14% of patients with single-positive anti-GBM disease received ongoing therapy (*P* < 0.001), of whom 80% received azathioprine and 20% received MMF.

### Outcomes

**Figure 2:**
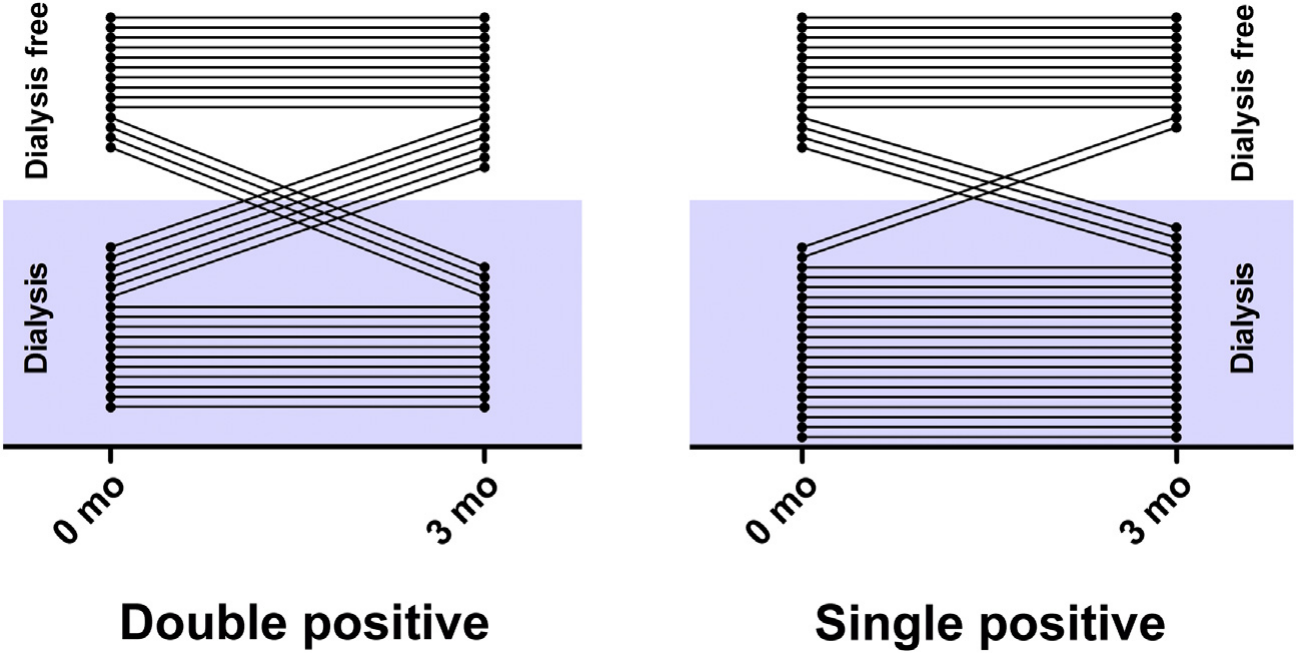
**Transition to and from dialysis dependence in the first 3 months (mo) following treatment, in double-positive and single-positive anti–glomerular basement membrane disease cases.** Censored for death in the first 3 months.

Patient and renal survival for all 3 cohorts at 3 and 12 months is summarized in Table 3. Overall patient survival was similar in all groups at both time points. Renal survival was favorable in the AAV group at both time points, although there was no significant difference in the proportion of patients who required dialysis in the anti-GBM and double-positive group at either time point. The proportion of patients who presented with dialysis-dependent renal failure and who recovered renal function and were alive at 1 year was significantly different between groups, varying from 17% in patients with single-positive anti-GBM disease to 29% in patients who were double positive and 49% in AAV cases. As Figure 2 demonstrates, this was due in part to cross-over from dialysis dependence to independence, and vice versa, particularly within the double-positive group, in which a substantial proportion recovered renal function in the first 3 months of follow-up (35% recovery vs. 10% recovery in patients who were single positive; *P* = 0.11). There was no significant difference in age (mean: 65 vs. 64 years; *P* = 0.88) or receipt of plasma exchange (100% vs. 82%; *P* = 0.52) between those patients and who were double-positive and who recovered and those who did not, respectively, although those who recovered renal function tended to have lower levels of anti-GBM antibodies (3.8 vs. 10 xULN, respectively; *P* = 0.07). Only one-half of the patients who were double-positive who recovered renal function underwent renal biopsy, and so it was not possible to reliably identify histopathological predictors of treatment response.

**Table 3:** Patient and renal survival at 3 and 12 months after diagnosis

Diagnosis	0 Months	3 months		12 months		Renal recovery at 1 year^b^
	Independent of RRT	Patient survival	Renal survival^a^	Patient survival	Renal survival^a^	
AAV	437/568 77%	540/568 95%	490/540 91%	512/568 90%	452/512 88%	64/131 49%
Anti-GBM	15/41 37%	37/41 90%	15/36 42%	36/41 87%	15/34 44%	4/24 17%
Double positive	16/37 43%	33/37 89%	16/32 50%	31/37 83%	16/30 53%	6/21 29%
*P* value	**<0.01**	0.13	**<0.01**	0.38	**<0.01**	**<0.01**

AAV, anti-neutrophil cytoplasm antibody–associated vasculitis; GBM, glomerular basement membrane; RRT, renal replacement therapy.

Comparison between groups by chi-square test.

a Censored for death.

b Proportion of patients requiring RRT at presentation who were alive with independent renal function at 1 year.

**Figure 3:**
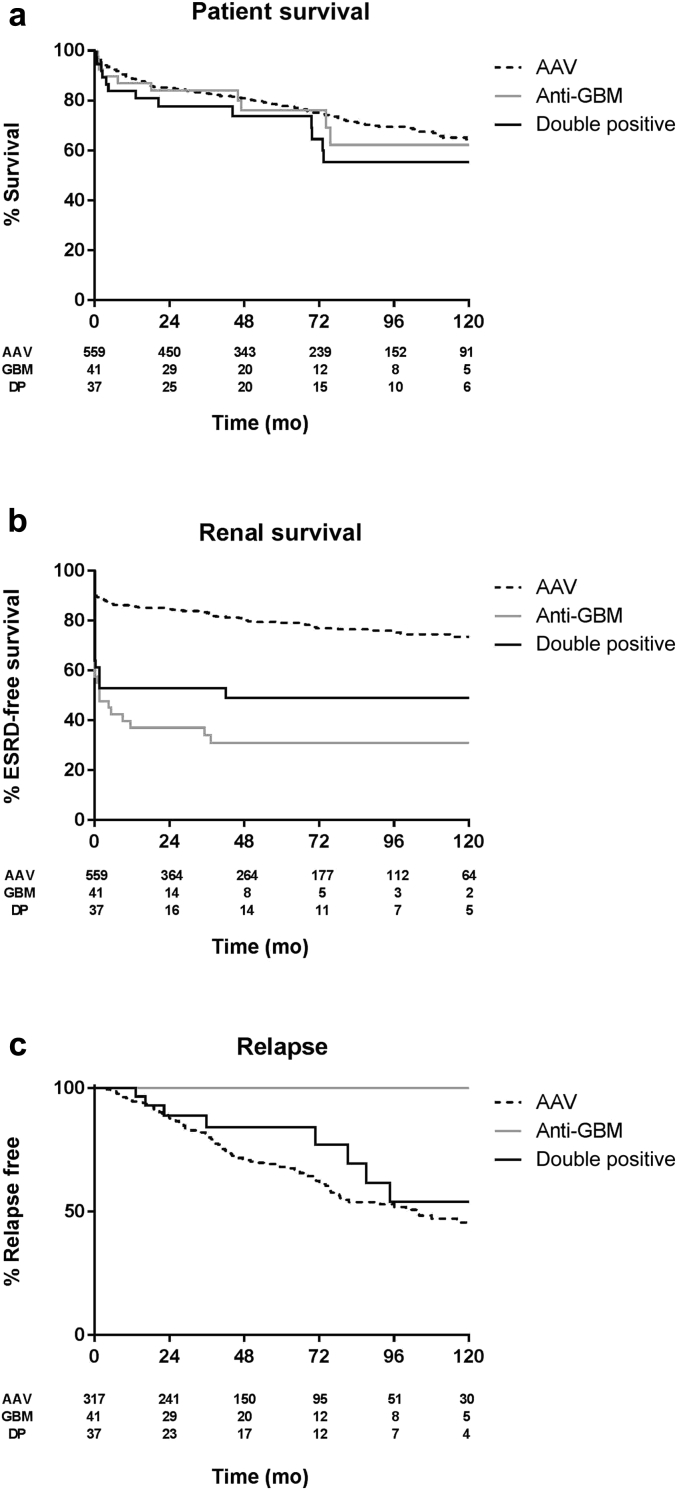
**Unadjusted Kaplan–Meier survival functions describing long-term patient, renal, and relapse-free survival rates of the study cohort during 10 years’ follow-up.** (**a**) Overall patient survival. (**b**) End-stage renal disease-free survival. (**c**) Relapse-free survival (censored for death). AAV, anti-neutrophil cytoplasm antibody–associated vasculitis; DP, double positive; ESRD, end-stage renal disease; GBM, glomerular basement membrane.

The median duration of follow-up was 4.8 years (range: 0–15 years). Long-term patient and renal survival is summarized in Figure 3a and b, respectively. No difference in unadjusted overall patient survival was observed during the study (*P* = 0.49). Renal survival was favorable in the AAV group compared with both the anti-GBM group (*P* < 0.01) and the double double-positive groups (*P* < 0.01). Patients who were double positive tended to have better renal survival than those who were single positive with anti-GBM disease, although this difference was not statistically significant in unadjusted analysis (*P* = 0.17).

### Relapse

Relapse data were available in 316 AAV patients and all patients with anti-GBM disease and double positivity until last follow-up. Within the follow-up period, 116 of 316 patients with AAV (37%) and 8 of 37 patients who were double positive (22%) had a relapse. Two patients with single-positive anti-GBM disease had early recrudescence of anti-GBM antibodies requiring augmented treatment in the first 6 months following initial diagnosis, which we believe reflected inadequate initial disease control rather than disease relapse. No patients with single-positive anti-GBM disease exhibited evidence of relapse or recurrent antibodies beyond 6 months. During long-term follow-up, a significant proportion (*n* = 8; approximately 50%) of the surviving patients who were double positive developed recurrent disease (Figure 3c). These relapses tended to occur late (median time to first relapse: 4.4 years; range: 1.1–7.9 years), and the majority (5 of 8) were in patients with ANCA directed against PR3, in keeping with the natural history of isolated AAV. Two patients had relapses associated with MPO-ANCA, and 1 notable patient had a relapse of both MPO-ANCA and anti-GBM antibodies. All relapses occurred in patient who were ANCA positive, and the majority (6 of 8) were associated with a rise in ANCA titer of more than 25%, or seroconversion from ANCA-negative to ANCA-positive status, in the 6 months prior to diagnosis of relapsing disease. Table 4 summarizes the clinical features of each relapse and its relation to immunosuppressive treatment. Notably, the majority of patients were not receiving maintenance treatment other than corticosteroids at the time of relapse. As shown in Figure 3c, the frequency of relapse in the double-positive cohort was comparable to that in AAV cases (*P* = 0.29), whereas there were no relapses in the anti-GBM group (*P* < 0.01). Two patients who were double positive have undergone renal transplantation. To date, no disease recurrence related to either ANCA or anti-GBM disease has been described in the renal allografts.

**Table 4:** Details of double-positive patients with relapse

Case	Time to relapse (months)	ANCA	GBM Ab	Organ involvement	Treatment at time of relapse	Treatment for relapse	Subsequent relapse
1	13	PR3	Neg	Renal, skin	CS only; CYC stopped 9 mo prior	CYC, CS	Yes
2	16	PR3	Neg	LRT	CS only; AZA stopped 4 mo prior	AZA, CS	No
3	22	MPO	Neg	LRT	CS only; AZA stopped 18 mo prior	CS	Yes
4	36	PR3	Neg	Renal, Skin	MMF, CS	RTX, CYC, CS	Yes
5	71	PR3	Neg	Renal	AZA	AZA, CS	No
6	81	PR3	Neg	Constitutional	CS only; AZA stopped 10 mo prior	AZA, CS	Yes
7	87	MPO	Neg	Renal	None; off treatment 5 yr	CS	No
8	95	MPO	Positive	LRT, Renal	None; off treatment 12 mo	RTX, CYC, CS	No

ANCA, anti-neutrophil cytoplasm antibody; AZA, azathioprine; CS, corticosteroids; CYC, cyclophosphamide; GBM, glomerular basement membrane; LRT, lower respiratory tract; MPO, myeloperoxidase; MMF, mycophenolate mofetil; mo, months; PR3, proteinase 3; RTX, rituximab; y, years.

### Predictors of death, ESRD, and relapse

**Figure 4:**
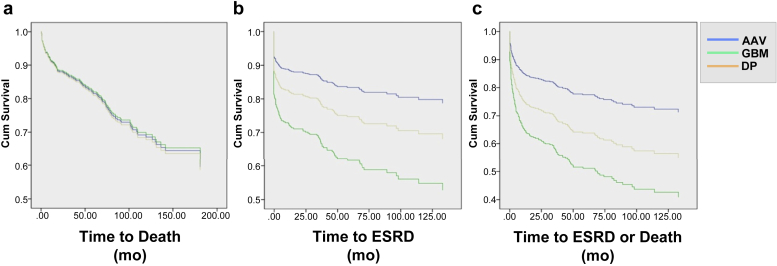
**Cox proportional hazards regression curves describing long-term risk of (a) death, (b) end-stage renal disease (ESRD), and (c) death or ESRD.** Measures being controlled for include diagnosis, age, requirement for renal replacement therapy at presentation, and presence of lung hemorrhage at presentation. AAV, anti-neutrophil cytoplasm antibody–associated vasculitis; DP, double positive; GBM, anti-glomerular basement membrane disease.

There were significant differences in age, proportion of patients requiring renal replacement therapy (RRT), and proportion of patients with lung hemorrhage at presentation between patients diagnosed with AAV, anti-GBM disease, and double positivity. We therefore performed regression analysis to identify predictors of death and end-stage renal disease (ESRD) in all patients, correcting for these differences in baseline variables (Table 5; Figure 4).

**Table 5:** Predictors of death and end-stage renal disease

Unadjusted analysis						
	Death		ESRD		Death or ESRD	
	HR (CI)	*P* value	HR (CI)	*P* value	HR (CI)	*P* value
Diagnosis	–	0.25	–	**<0.01**	–	**<0.01**
DP^a^ versus AAV	0.72 (0.406–1.26)	0.25	0.31 (0.19–0.51)	**<0.01**	0.33 (0.42–0.52)	**<0.01**
DP^a^ versus GBM	0.78 (0.34–1.79)	0.56	1.542 (0.85–2.80)	0.16	1.38 (0.78–2.45)	0.27
AAV^a^ versus GBM	1.10 (0.58–2.08)	0.78	4.98 (3.25–7.64)	**<0.01**	4.21 (2.78–6.37)	**<0.01**
Age at presentation	1.05 (1.04–1.06)	**<0.01**	1.01 (0.99–1.02)	0.18	1.01 (1.00–1.02)	**0.01**
RRT at presentation	2.20 (1.63–2.97)	**<0.01**	9.34 (6.53–13.33)	**<0.01**	5.71 (4.17–15.38)	**<0.01**
LH at presentation	1.45 (1.06–1.99)	**0.02**	1.89 (1.36–2.63)	**<0.01**	1.79 (1.32–2.38)	**<0.01**

Multivariable analysis						
	Death		ESRD		Death or ESRD	
	HR (CI)	*P* value	HR (CI)	*P* value	HR (CI)	*P* value
Diagnosis	–	0.95		**<0.01**	–	**<0.01**
DP^a^ versus AAV	0.97 (0.52–1.81)	0.92	0.62 (0.36–1.06)	0.08	0.57 (0.35–0.93)	**0.02**
DP^a^ versus GBM	0.94 (0.39–2.28)	0.89	1.66 (0.88–3.12)	0.12	1.49 (0.82–2.72)	0.19
AAV^a^ versus GBM	0.97 (0.48–1.95)	0.93	2.66 (1.69–4.19)	**<0.01**	2.63 (1.69–4.09)	**<0.01**
Age at presentation	1.05 (1.04–1.06)	**<0.01**	1.01 (1.00–1.02)	0.11	1.02 (1.01–1.03)	**0.03**
RRT at presentation	2.04 (1.49–2.78)	**<0.01**	7.69 (5.26–11.1)	**<0.01**	4.55 (3.33–6.25)	**<0.01**
LH at presentation	1.37 (0.99–1.89)	0.06	1.15 (0.82–1.61)	0.42	1.21 (0.88–1.64)	0.23

AAV, anti-neutrophil cytoplasm antibody–associated vasculitis; DP, double positive; ESRD, end-stage renal disease; GBM, anti–glomerular basement membrane disease; HR, hazard ratio; LH, lung hemorrhage; RRT, renal replacement therapy.

a Reference group for estimates of hazard ratios.

Unadjusted predictors of death included RRT at presentation (hazard ratio [HR]: 2.2 [1.63–2.97]; *P* < 0.01), lung hemorrhage (HR: 1.45 [1.06–1.99]; *P* = 0.02), and age (HR: 1.05 [1.04–1.06]; *P* < 0.01), but not diagnosis. In multivariable analysis, RRT at presentation (HR: 2.04 [1.49–2.78], *P* < 0.01) and age (HR: 1.05 [1.04–1.06]; *P* < 0.01) predicted death. The influence of lung hemorrhage at presentation (HR: 1.37 [0.99–1.89]; *P* = 0.06) approached statistical significance, while diagnosis had no influence.

Unadjusted predictors of progression to ESRD included diagnosis (*P* < 0.01), lung hemorrhage at presentation (HR: 1.89 [1.32–2.63]; *P* < 0.01), and RRT at presentation (HR: 9.34 [6.53–13.33]; *P* < 0.01). Age was not associated with progression to ESRD (*P* = 0.18). In multivariable analysis, RRT at presentation (HR: 7.69 [5.26–11.10]; *P* < 0.01) and diagnosis (*P* < 0.01) increased the HR of progression to ESRD. The risk of ESRD was increased in anti-GBM disease compared with AAV (HR: 2.66 [1.69–4.19], *P* < 0.01), though the risk in patients who were double positive versus those with AAV was not significantly different (HR: 0.62 [0.36–1.06]; *P* = 0.08), suggesting an intermediate risk of ESRD in patients who were double positive. Age (*P* = 0.11) and lung hemorrhage at presentation (*P* = 0.42) had no influence on progression to ESRD.

Unadjusted predictors of a composite outcome of death or progression to ESRD included age (HR: 1.01 [1.00–1.02]; *P* = 0.01), diagnosis (*P* < 0.01), RRT at presentation (HR: 5.71 [4.17–15.38]; *P* < 0.01), and lung hemorrhage at presentation (HR: 1.79 [1.08–2.94]; *P* < 0.01). In multivariable analysis, diagnosis (*P* < 0.01), RRT at presentation (HR: 4.55 [3.33– 6.25]; *P* < 0.01), and age (HR: 1.02 [1.01–1.03]; *P* < 0.01) were associated with progression to death or ESRD. Lung hemorrhage at presentation was not associated with progression to death or ESRD (*P* = 0.23). Death or progression to ESRD was similar between double-positive and single-positive anti-GBM disease cases. However, patients with AAV had a lower hazard ratio of progression to ESRD or death compared with patients who were double positive (HR: 0.57 [0.35–0.93]; *P* = 0.02).

Unadjusted predictors of relapse included diagnosis (*P* < 0.01), and RRT dependence at presentation was associated with a lower risk of relapse (*P* = 0.02). Further analysis was not conducted due to the relative small number of relapse episodes.

## Discussion

This is the largest published series to compare the outcomes of patients with both ANCA and anti-GBM autoantibodies with patients with single-positive AAV and anti-GBM disease. As such, it provides several important observations: the phenomenon of double positivity is common, these patients experience the early morbidity and mortality typical of anti-GBM disease, and they carry the long-term risk of relapse typical of AAV.

Patients who are double positive accounted for approximately one-half of all anti-GBM disease cases seen at our centers since 2000, and over 10% of AAV patients with renal involvement seen at the UK site over the same time period. The proportion of AAV cases was variable at the other sites, perhaps reflecting differences in referral pattern at each (with varying proportions of patients with extrarenal vasculitis) and differences in sensitivity of assays used to detect ANCA, or geographical variations in disease frequency. Notably, a recent study reported that more than 60% of patients with anti-GBM disease had autoantibodies reactive to linear epitopes of MPO, versus 24% who had antibodies to native protein detected by conventional assays. Our study highlights this common concurrence, and our observations reiterate the need to determine the alternative antibody type in all cases of AAV- or anti-GBM disease.

In this large series, we observed comparable severity of disease at presentation between single-positive anti-GBM and double-positive cases, with approximately 60% of patients requiring renal replacement therapy at presentation, and one-third developing lung hemorrhage, in both groups. Regression analysis suggests that it is these severe disease manifestations, rather than diagnosis *per se*, that affect overall patient survival, and because they are equally prevalent in patients with anti-GBM disease and those who are double positive (and less frequent than in AAV), this suggests that anti-GBM disease is the “dominant” early disease phenotype in double-positive cases. These patients, however, also demonstrate clinical features of AAV, such as an older age distribution, a longer prodrome of systemic symptoms, and features of chronic damage on renal biopsy (in excess of what would be expected for the age difference between groups). In addition, regression analysis suggests that patients who are double positive have an intermediate risk of progression to ESRD compared with single-positive AAV or anti-GBM cases. This may be related to the observation that more than one-third of the surviving patients who were double positive and required dialysis at presentation regained independent renal function by 3 months. This propensity to renal recovery was more in keeping with AAV than anti-GBM disease, where regaining renal function from dialysis is very uncommon,^17^ and consistent with some previous reports of double-positive cases.^4^, ^10^, ^11^

That our patients who were double positive had this tendency to renal recovery and intermediate long-term renal survival, despite more chronic renal damage on kidney biopsy and more advanced age at presentation, is striking. Responders tended to have lower levels of anti-GBM antibodies, suggesting that they may have been identified early in the course of anti-GBM disease, or that they may have a “milder” form of disease. Of note, there are several recent reports of “atypical” variants of anti-GBM disease, each characterized by less severe renal involvement than is usually observed.^18^, ^19^, ^20^ Differences in antibody subclass or in antigen or epitope specificity may account for these variable presentations. With regard to patients who are double positive, 1 previous study found that they had a broader spectrum of anti-GBM antibodies and lower reactivity to a3(IV)NC1 than patients who were single positive,^21^ although an earlier study did not report differences in antigen specificity.^22^ It is therefore possible that differences in antigen or epitope specificity account for the difference in treatment response seen in our cohort; however, we were unable to analyze this in detail in a retrospective study. Likewise, we have been unable to identify histopathological predictors of recovery, a significant limitation of our analysis. Previous studies have shown that the proportion of crescentic and normal glomeruli in anti-GBM disease is predictive of prognosis,^17^, ^23^ and a prognostic classification based on histopathological findings has been proposed for ANCA-associated glomerulonephritis.^24^, ^25^ It is unclear, however, whether these observations apply to patients who are double positive. Given the overall rarity of anti-GBM disease, a larger, multicenter analysis of histopathological lesions in these cases is likely needed in order to infer reliable prognostic indicators. Pending such information, our observations suggest that while patients who are double positive behave primarily like those with isolated anti-GBM disease, a subset of patients who are dialysis dependent at presentation may be more responsive to therapy, and aggressive treatment may be warranted in some cases.

The other striking characteristic of AAV retained by patients who are double positive is a risk of disease relapse. The long-term follow-up offered by our study suggests that almost one-half of surviving patients who are double positive will experience disease relapse at some point, at a frequency comparable to that observed in our single-positive AAV cohort. As might be expected, these relapses were more likely in patients who were anti-PR3 positive, and were associated with preceding increases in ANCA titer. Of note, 1 patient relapsed with both ANCA and anti-GBM antibodies. These observations suggest that while the dominant early disease phenotype in these patients is anti-GBM disease, unlike patients with isolated anti-GBM disease these cases require frequent long-term follow-up and consideration of maintenance immunosuppression. That 1 of our patients had an earlier diagnosis of isolated AAV prior to presenting with a double-positive “relapse” also suggests that anti-GBM antibodies should be determined in relapsing AAV cases, particularly if there is evidence of renal involvement.

The mechanism of association between AAV and anti-GBM disease in unclear. Studies in animal models suggest that administration of the alternate antibody type may augment the severity of renal disease in models of either vasculitis or anti-GBM nephritis;^26^, ^27^, ^28^ however, these *in vivo* studies have shed little light on the spontaneous development of both antibody types, or on the sequence in which they develop in clinical disease. An elegant clinical study by Olson and colleagues, using stored sera from the US Department of Defense, suggested that the majority of patients with anti-GBM disease had detectable ANCA prior to the development of anti-GBM antibodies, which in turn predated the development of clinical disease, suggesting that AAV may act as trigger for anti-GBM disease.^29^ Our observations support this hypothesis: patients who were double positive had the age restriction of isolated AAV cases, a longer prodrome of systemic symptoms prior to diagnosis, and more features of chronicity on their renal biopsy, suggesting that ANCA-mediated glomerular inflammation may precede and contribute to the development of anti-GBM disease, perhaps by disrupting the quaternary structure of the GBM.^30^ This could lead to the exposure of normally sequestered epitopes in a pro-inflammatory milieu, resulting in a fulminant anti-GBM response. Conversely, it has been shown that aberrant extracellular expression of myeloperoxidase, as a constituent of neutrophil extracellular traps, may predispose to the development of anti-MPO antibodies,^31^ and that neutrophil extracellular traps are formed in experimental anti-GBM disease.^32^ Thus, it is possible that glomerular neutrophil recruitment and activation in anti-GBM disease similarly contributes to the development of ANCA. The recent observation that a high proportion of patients with anti-GBM disease have autoantibodies reactive to linear epitopes of MPO might support this hypothesis, as it suggests reactivity to conformational MPO epitopes might arise as a consequence of inter- and intramolecular epitope spreading initiated by anti-GBM disease.^33^ Whether additional environmental or genetic factors predispose to forming both antibodies is unclear. The genetic associations of both anti-GBM disease and AAV are increasingly well-described,^34^, ^35^ and both conditions have strong associations with certain HLA genes. Notably, both conditions have reported associations with HLA-DRB1*1501, and a previous small study observed a DRB1-15 genotype in 4 of the 5 patients who were double positive that were examined.^36^

In this descriptive, retrospective study, we have been unable to analyze the genetic or detailed serological and pathological features of our cohort. Strengths of our study, however, include its large size, its long follow-up period beyond 10 years for many patients, the inclusion of all single-positive anti-GBM and AAV cases by way of controlled comparison, and that it is multicenter, from international sites that utilize comparable treatment regimens. We highlight several important clinical practice points—in particular that while anti-GBM disease is the predominant disease phenotype in these patients, their ANCA status should neither be ignored nor forgotten, because a subset may be more responsive to initial immunosuppressive treatment, and they have a significant risk of relapse requiring careful long-term follow-up and monitoring.

## Methods

This is a retrospective analysis of patients diagnosed with AAV, anti-GBM disease, and double-positive ANCA and anti-GBM antibody disease from 4 European centers: Hammersmith Hospital, London, UK; Charles University Hospital, Prague, Czech Republic; Skånes University Hospital, Lund, Sweden; and Linköping University Hospital, Linköping, Sweden. All patients diagnosed between 2000 and 2013 with at least 1 year of follow-up were included in analysis.

Patients with a diagnosis of systemic vasculitis consistent with the Chapel Hill Consensus Conference^37^ and positive ANCA serology were included in the AAV group. Anti-GBM disease was defined by either (i) the presence of circulating anti-GBM antibodies in association with clinical manifestations of alveolar hemorrhage and/or rapidly progressive glomerulonephritis, or (ii) biopsy-proven crescentic glomerulonephritis with linear deposition of IgG along the GBM in the absence of another attributable cause (such as diabetes mellitus or paraproteinaemia). The double-positive cohort included patients who met this diagnosis of anti-GBM disease and in addition had positive ANCA serology.

Circulating anti-GBM antibodies were identified using conventional commercially available assays, which varied between site and over time at each center. Antigen substrates included purified bovine or human GBM preparations and recombinant human α3(IV) collagen chain. ANCA was detected either by indirect immunofluorescence using ethanol–fixed human neutrophils, or by antigen specific assays using commercially available ELISA or bead-based multiplex assays, which used purified human ANCA antigens. Patients who are ANCA positive were subclassified by ANCA specificity to either myeloperoxidase or PR3 antigens. In patients who tested positive by fluorescence testing but negative by antigen-specific assay, those with a perinuclear indirect immunofluorescence pattern were assigned to the MPO group and those with a cytoplasmic pattern to the PR3 group.

Following identification of cases, the case notes, pathology, and laboratory records were reviewed to collect data using an electronic database on details of clinical presentation, treatment, and outcomes. Patients were followed up from presentation until last clinical encounter prior to December 31, 2014. RRT at presentation was defined by the need for acute dialysis during the first hospital admission. ESRD was defined by a sustained requirement for RRT that did not recover during follow-up or before death. GFR was estimated by the Modified Diet in Renal Disease calculation.^38^ Relapses were defined by an increase or recurrence in disease activity requiring augmented immunotherapy. For histopathological analysis, we reviewed original renal biopsy reports. We defined “crescentic” glomeruli by the presence of cellular, fibrocellular, or fibrous crescents. Synchronous crescent formation was defined by the presence of uniformly aged glomerular crescents in the biopsy, whereas a mix of cellular, fibrocellular, or fibrous crescents defined “asynchronous” crescent formation. Obsolete glomeruli, and those with segmental scars, were included in the category of “sclerotic” glomeruli. “Normal” glomeruli included those with minor mesangial or ischemic changes only, without significant proliferation, scarring, or crescent formation.

We compared baseline clinical features and long-term outcomes between all 3 diagnoses. In addition, we performed more detailed comparison of histopathology and treatment in the single-positive anti-GBM and double-positive groups. Continuous data were regarded as nonparametric. For comparison of continuous variables, Mann-Whitney (2 groups) and Kruskal–Wallis with *post hoc* Dunn’s test (more than 2 groups) were used to ascertain differences between individual groups. For comparison of categorical variables between groups, chi-square test was used. Log-rank test was used to ascertain unadjusted survival differences and plotted as Kaplan–Meier curves. Cox proportional regression analysis was used to ascertain proportional hazards ratio of factors associated with categorical outcomes (death, ESRD progression, and death or ESRD progression as a composite outcome). Covariates included in the multivariable Cox proportional regression analysis included diagnosis, age, RRT at presentation, and lung hemorrhage at presentation. For the diagnosis subgroup in the Cox regression analyses, diagnosis was entered into the model as a categorical predictor, with double-positive chosen as the reference. The analysis was repeated with AAV as the reference subgroup to evaluate differences between AAV and anti-GBM disease. Proportionality assumption was met for covariates included in the Cox regression analysis. Data are presented as hazards ratios (confidence interval; *P* value). Graphs were constructed and statistical analysis performed using Prism 5.0 (GraphPad Software, La Jolla, CA) and SPSS version 22.0 (IBM Corp., Armonk, NY).

As this was a retrospective study and all treatment decisions were made prior to our assessment, research ethics approval was not required for this report.

## Disclosure

All the authors declared no competing interests.

## References

[1] Pusey C.D. (2003). Anti-glomerular basement membrane disease. Kidney Int.

[2] Watts R.A., Mahr A., Mohammad A.J. (2015). Classification, epidemiology and clinical subgrouping of antineutrophil cytoplasmic antibody (ANCA)-associated vasculitis. Nephrol Dial Transplant.

[3] O’Donoghue D.J., Short C.D., Brenchley P.E. (1989). Sequential development of systemic vasculitis with anti-neutrophil cytoplasmic antibodies complicating anti-glomerular basement membrane disease. Clin Nephrol.

[4] Jayne D.R., Marshall P.D., Jones S.J. (1990). Autoantibodies to GBM and neutrophil cytoplasm in rapidly progressive glomerulonephritis. Kidney Int.

[5] Levy J.B., Hammad T., Coulthart A. (2004). Clinical features and outcome of patients with both ANCA and anti-GBM antibodies. Kidney Int.

[6] Rutgers A., Slot M., van Paassen P. (2005). Coexistence of anti-glomerular basement membrane antibodies and myeloperoxidase-ANCAs in crescentic glomerulonephritis. Am J Kidney Dis.

[7] Zhao J., Yang R., Cui Z. (2007). Characteristics and outcome of Chinese patients with both antineutrophil cytoplasmic antibody and antiglomerular basement membrane antibodies. Nephron Clin Pract.

[8] J DEZ, Taylor D., Thein H. (2011). Incidence and features of dual anti-GBM-positive and ANCA-positive patients. Nephrology.

[9] Short A.K., Esnault V.L., Lockwood C.M. (1995). Anti-neutrophil cytoplasm antibodies and anti-glomerular basement membrane antibodies: two coexisting distinct autoreactivities detectable in patients with rapidly progressive glomerulonephritis. Am J Kidney Dis.

[10] Bosch X., Mirapeix E., Font J. (1991). Prognostic implication of anti-neutrophil cytoplasmic autoantibodies with myeloperoxidase specificity in anti-glomerular basement membrane disease. Clin Nephrol.

[11] Segelmark M., Hellmark T., Wieslander J. (2003). The prognostic significance in Goodpasture's disease of specificity, titre and affinity of anti-glomerular-basement-membrane antibodies. Nephron Clin Pract.

[12] Weber M.F., Andrassy K., Pullig O. (1992). Antineutrophil-cytoplasmic antibodies and antiglomerular basement membrane antibodies in Goodpasture’s syndrome and in Wegener's granulomatosis. J Am Soc Nephrol.

[13] Lindic J., Vizjak A., Ferluga D. (2009). Clinical outcome of patients with coexistent antineutrophil cytoplasmic antibodies and antibodies against glomerular basement membrane. Ther Apher Dial.

[14] Srivastava A., Rao G.K., Segal P.E. (2013). Characteristics and outcome of crescentic glomerulonephritis in patients with both antineutrophil cytoplasmic antibody and anti-glomerular basement membrane antibody. Clin Rheumatol.

[15] Alchi B., Griffiths M., Sivalingam M. (2015). Predictors of renal and patient outcomes in anti-GBM disease: clinicopathologic analysis of a two-centre cohort. Nephrol Dial Transplant.

[16] Cui Z., Zhao J., Jia X.Y. (2011). Anti-glomerular basement membrane disease: outcomes of different therapeutic regimens in a large single-center Chinese cohort study. Medicine (Baltimore).

[17] Levy J.B., Turner A.N., Rees A.J., Pusey C.D. (2001). Long-term outcome of anti-glomerular basement membrane antibody disease treated with plasma exchange and immunosuppression. Ann Intern Med.

[18] McAdoo S.P., Tanna A., Randone O. (2015). Necrotizing and crescentic glomerulonephritis presenting with preserved renal function in patients with underlying multisystem autoimmune disease: a retrospective case series. Rheumatology (Oxford).

[19] Ohlsson S., Herlitz H., Lundberg S. (2014). Circulating anti-glomerular basement membrane antibodies with predominance of subclass IgG4 and false-negative immunoassay test results in anti-glomerular basement membrane disease. Am J Kidney Dis.

[20] Nasr S.H., Collins A.B., Alexander M.P. (2016). The clinicopathologic characteristics and outcome of atypical anti-glomerular basement membrane nephritis. Kidney Int.

[21] Yang R., Hellmark T., Zhao J. (2007). Antigen and epitope specificity of anti-glomerular basement membrane antibodies in patients with goodpasture disease with or without anti-neutrophil cytoplasmic antibodies. J Am Soc Nephrol.

[22] Hellmark T., Niles J.L., Collins A.B. (1997). Comparison of anti-GBM antibodies in sera with or without ANCA. J Am Soc Nephrol.

[23] Fischer E.G., Lager D.J. (2006). Anti-glomerular basement membrane glomerulonephritis: a morphologic study of 80 cases. Am J Clin Pathol.

[24] Berden A.E., Ferrario F., Hagen E.C. (2010). Histopathologic classification of ANCA-associated glomerulonephritis. J Am Soc Nephrol.

[25] Tanna A., Guarino L., Tam F.W. (2015). Long-term outcome of anti-neutrophil cytoplasm antibody-associated glomerulonephritis: evaluation of the international histological classification and other prognostic factors. Nephrol Dial Transplant.

[26] Kobayashi K., Shibata T., Sugisaki T. (1995). Aggravation of rat nephrotoxic serum nephritis by anti-myeloperoxidase antibodies. Kidney Int.

[27] Heeringa P., Brouwer E., Klok P.A. (1996). Autoantibodies to myeloperoxidase aggravate mild anti-glomerular-basement-membrane-mediated glomerular injury in the rat. Am J Pathol.

[28] Kanzaki G., Nagasaka S., Higo S. (2016). Impact of anti-glomerular basement membrane antibodies and glomerular neutrophil activation on glomerulonephritis in experimental myeloperoxidase-antineutrophil cytoplasmic antibody vasculitis. Nephrol Dial Transplant.

[29] Olson S.W., Arbogast C.B., Baker T.P. (2011). Asymptomatic autoantibodies associate with future anti-glomerular basement membrane disease. J Am Soc Nephrol.

[30] Pedchenko V., Bondar O., Fogo A.B. (2010). Molecular architecture of the Goodpasture autoantigen in anti-GBM nephritis. The New England journal of medicine.

[31] Nakazawa D., Tomaru U., Suzuki A. (2012). Abnormal conformation and impaired degradation of propylthiouracil-induced neutrophil extracellular traps: implications of disordered neutrophil extracellular traps in a rat model of myeloperoxidase antineutrophil cytoplasmic antibody-associated vasculitis. Arthritis Rheum.

[32] Kumar S.V., Kulkarni O.P., Mulay S.R. (2015). Neutrophil extracellular trap-related extracellular histones cause vascular necrosis in severe GN. J Am Soc Nephrol.

[33] Li J.N., Cui Z., Wang J. (2016). Autoantibodies against linear epitopes of myeloperoxidase in anti-glomerular basement membrane disease. Clin J Am Soc Nephrol.

[34] Rahmattulla C., Mooyaart A.L., van Hooven D. (2016). Genetic variants in ANCA-associated vasculitis: a meta-analysis. Ann Rheum Dis.

[35] Zhou X.J., Lv J.C., Zhao M.H. (2010). Advances in the genetics of anti-glomerular basement membrane disease. Am J Nephrol.

[36] Persson U., Hertz J.M., Carlsson M. (2004). Patients with Goodpasture’s disease have two normal COL4A3 alleles encoding the NC1 domain of the type IV collagen alpha 3 chain. Nephrol Dial Transplant.

[37] Jennette J.C., Falk R.J., Bacon P.A. (2013). 2012 revised International Chapel Hill Consensus Conference Nomenclature of Vasculitides. Arthritis Rheum.

[38] Levey A.S., Bosch J.P., Lewis J.B. (1999). A more accurate method to estimate glomerular filtration rate from serum creatinine: a new prediction equation. Modification of Diet in Renal Disease Study Group. Ann Intern Med.

